# Bilateral and Symmetrical Lesions in the Basal Ganglia Associated With Metabolic Acidosis in a Patient With a History of Alcohol Addiction: A Case Report

**DOI:** 10.7759/cureus.26307

**Published:** 2022-06-24

**Authors:** André E Almeida Franzoi, Carolina F Colaço, Luis E Borges de Macedo Zubko, Matheus F Nascimento de Souza, Rodrigo S Kruger

**Affiliations:** 1 Neurology Department, Hospital de Clínicas de Curitiba (Federal University of Paraná), Curitiba, BRA; 2 Medicine Department, University of Joinville Region (UNIVILLE), Joinville, BRA; 3 Intensive Care Department, Hospital de Clínicas de Curitiba (Federal University of Paraná), Curitiba, BRA

**Keywords:** methanol poisoning, high anion gap metabolic acidosis, methanol, addiction, basal ganglia

## Abstract

Metabolic acidosis is defined as a pathologic process that, when unopposed, increases the concentration of hydrogen ions in the body and reduces the concentration of HCO3. Methanol poisoning is an important cause of metabolic acidosis. Methanol and ethylene glycol poisonings cause scores of fatal intoxications annually, and even relatively small ingestions of these alcohols can produce significant toxicity. Neuroimaging findings are very suggestive and help in the diagnosis even before the measurement of serum methanol (when available at the health service). Rapid recognition and early treatment, including alcohol dehydrogenase inhibition, are crucial. In this sense, some studies question that many intoxications by different chemical agents (in addition to methanol and ethylene glycol) generate a conglomeration of neuroimaging findings that summarily reflect the presence of metabolic acidosis. Therefore, in this article, we discuss the imaging findings of metabolic acidosis, methanol poisoning, and their main differential diagnoses in neuroimaging, directing earlier diagnostic reasoning in order to initiate the most appropriate treatment promptly.

## Introduction

Bilateral and symmetrical lesions in the basal ganglia can manifest as intoxication, CNS infections, neoplasms, liver diseases, metabolic diseases, and other clinical disorders. Methanol poisoning is an important cause of basal ganglia lesions and is highly suggestive in cases of alcoholism and metabolic acidosis history [1].

Methanol is a simple liquid alkanol that can be easily mistaken for ethanol [2]. It is highly toxic (1 g/kg can be the lethal dose) and unsuitable for human consumption [2,3], unlike ethanol. Methanol poisoning is rare, being reported mainly in the context of suicidal or accidental oral ingestion of methanol-containing agents or consumption of adulterated alcoholic beverages [2,3].

Patients can present several disabling clinical features. The main clinical manifestations are visual disturbances, mainly optic neuritis and eventual blindness [1,2]; CNS deficits, such as headache, neurological dizziness, fatigue, and eventual permanent dysfunction [1-3]; gastrointestinal involvement, such as nausea, vomiting, and abdominal pain [2,4]; severe metabolic acidosis with a high anion gap and without compensation [2-4]; and eventual coma and death [2,3]. These symptoms often present after a 12-hour latency.

## Case presentation

**Figure 1:**
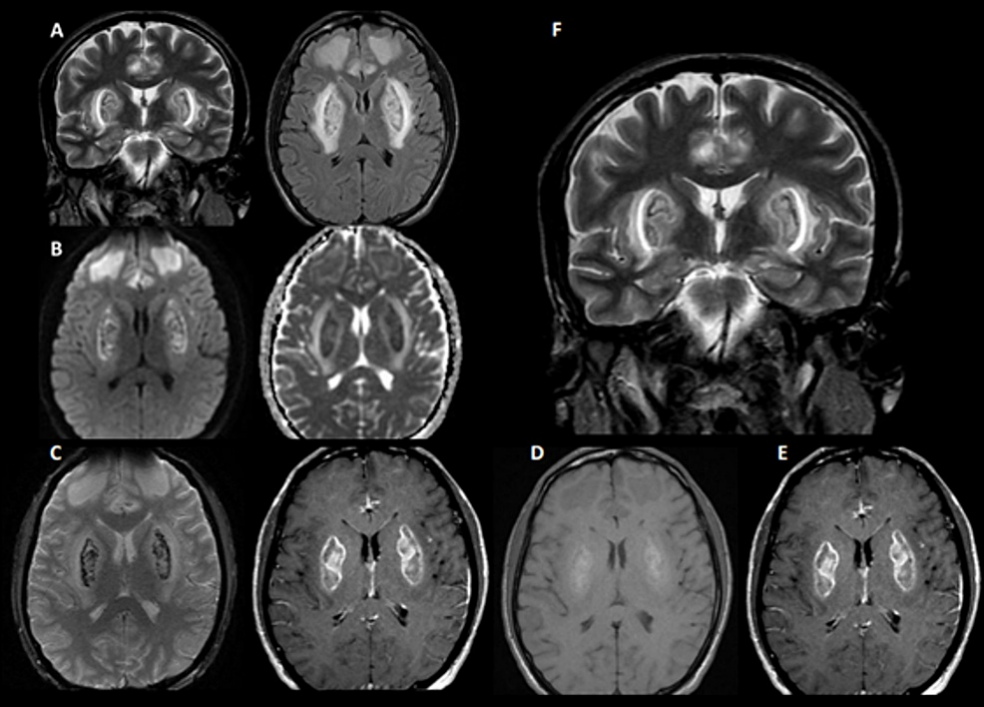
MRI with bilateral and symmetrical basal ganglia lesions. A) Areas of hyperintensity in T2 and T2 FLAIR deep white substance of the frontal lobes, anterior portions of the cingulate/medial region of the frontal lobes, and striated bodies. B) Restriction to the diffusion of water in DWI (hypersignal in DWI and hyposignal in ADC) symmetrically affecting the deep white substance of the frontal lobes, anterior portions of the cingulate/medial region of the frontal lobes, and corpus striatum. C) With the exception of the deep white matter of the frontal lobes, the other lesions had foci of low signal on T2* and high signal on T1, suggesting hemorrhage. D) T1 pre-contrast image. E) T1 post-contrast image showing predominantly peripheral enhancement by contrast around the lesions in the basal ganglia, bilaterally. Leptomeningeal enhancement was observed adjacent to the cingulate gyri and the medial regions of the frontal lobes. F) Visualization of the coronal T2 with hypersignals affecting the symmetrical corpus striatum. T1: T1-weighted pulse sequence; T2: T2-weighted pulse sequence; T2*: echo gradient weighting; FLAIR, fluid-attenuated inversion recovery; DWI: diffusion-weighted imaging; ADC: apparent diffusion coefficient

A 30-year-old male with a 20-year history of chronic alcoholism, cocaine, and crack was admitted to the emergency department after a suicide attempt with symptoms of headache, dyspnea, nausea, vomiting, mental confusion, psychomotor agitation, and a lowering of the level of consciousness. After stabilizing his vital functions, the laboratory test results indicated metabolic acidosis with an increased anion gap, and the patient was referred to the ICU. After five days of hospitalization, magnetic resonance imaging (MRI) was performed, which indicated bilateral and symmetrical hyperintensities in the basal ganglia and bilateral frontal white matter, in addition to perilesional necro-hemorrhagic foci (Figure 1).

The patient was treated with intravenous replacement of HCO3 and ethanol. There was a partial clinical improvement within two weeks. In the internal medicine infirmary, the patient had deficits: dysmetria, dysarthria, and appendicular tremor in the upper limbs. The patient was discharged for outpatient follow-up. After one semester, in an outpatient medical evaluation, the patient presented partial clinical improvement but still maintained dysmetria, dysarthria, and appendicular tremor in the upper limbs.

## Discussion

Due to differences in its metabolism, methanol exhibits increased toxicity compared to ethanol. Methanol is metabolized to formaldehyde by the enzyme alcohol dehydrogenase [4]. Formaldehyde is metabolized into formate (formic acid) by the enzyme aldehyde dehydrogenase [4]. At this point, the format is a highly toxic respiratory chain metabolism toxin, as this toxin inhibits cytochrome oxidase. This action can generate cell hypoxia, resulting in necrosis, metabolic acidosis, and optic nerve demyelination [4]. It is hypothesized that the putamen is greatly affected by this process due to its high metabolic demands [2]. As this metabolic process evolves, there is a latency period from 12 to 24 hours before the main clinical manifestations appear [2,4].

The reported clinical case portrayed shows neuroimaging features suggestive of methanol intoxication. The metabolic acidosis generated by this intoxication is proposed as the mainstay of brain injuries. This poisoning tends to generate bilateral and symmetrical lesions. Thus, the major neuroimaging marks are lesions in the putamen, optic nerves, and retina, which may also affect the basal ganglia nuclei, subcortical white matter, and cerebellum [1,2]. Typically, these areas denote hyperintensity in the T2 and T2 FLAIR pulse sequences [2,3].

Depending on the degree of necrosis and demyelination, during the chronic phase, cystic cavities may develop in the putamen [2]. However, it is important to consider the physiological changes in the basal ganglia, such as the Virchow-Robin space and bilateral physiological calcifications [5], and other lesion factors in the basal ganglia as differential diagnoses, as shown in Table 1.

**Table 1:** Differential diagnosis of lesions in the basal ganglia.

Differential diagnosis		References
Toxic poisoning	Carbon monoxide, methanol, and cyanide are cellular respiratory toxins that affect the mitochondria. Thus, they reach brain areas with intense metabolic activity, such as the basal ganglia region.	[6,7]
Liver disease	Acute hyperammonemia, mainly occurring in cirrhotic patients with acute decompensated hepatic failure, can generate involvement with T2 and DWI denoting bilaterally symmetric swelling, hyperintensity, and restricted diffusion in the caudate heads, putamen, and insular cortices.	[8,9]
Nonketotic hyperglycemia	CT typically shows bilateral pallidal and caudate hyperattenuating. At MRI, the abnormal areas are hyperintense on T1 and of variable intensity on T2.	[10]
Hypoglycemia	The characteristic MRI findings are bilateral T2 hyperintensity in the cerebral cortex, hippocampus, and basal ganglia.	[11]
Hypoxic-ischemic encephalopathy (HIE)	HIE in patients who were resuscitated after cardiorespiratory arrest may suffer brain lesions denoted on T2 MRI with symmetric hyperintense bilateral areas in the basal ganglia, thalamus, and cerebral cortex.	[12]
Mitochondrial diseases	The highlight is Leigh disease with MRI findings of symmetric areas of T2 hypersignal in the basal ganglia (with putaminal involvement), periaqueductal region, and cerebral peduncles.	[13]
Wilson disease	MRI with T2 hyperintensity in the caudate nuclei, putamen, globus pallidus, and thalamus. Thalamic involvement usually in the ventrolateral portion. The cortical and subcortical regions, mesencephalon, pons, vermis, and dentate nuclei may also be involved. DWI restriction is often seen in the early stages of the disease.	[14,15]
Osmotic demyelination syndrome	In central pontine myelinolysis, MRI denotes a symmetric trident-shaped or bat wing-shaped area of hyperintensity in the central pons highlighted on T2 and T2 FLAIR. The ventrolateral pons and the pontine portion of the corticospinal tracts tend to be uninjured. Extrapontine myelinolysis can generate areas of T2 hypersignal in the putamen, globus pallidus, thalamus, and cerebellum.	[16]
Neurodegeneration with brain iron accumulation (NBIA)	MRI with bilateral hypointensity in the globus pallidus at T2 sequence. Patients with pantothenate kinase-associated neurodegeneration (PANK) with the PANK2 mutation demonstrate the “eye-of-the-tiger sign,” with a center of hyperintensity surrounded by the more typical hypointensity in the globus pallidus. The eye-of-the-tiger sign is not seen in PANK2 mutation-negative patients.	[17]
Fahr disease	Noncontrast CT scan showing bilaterally symmetrical high-attenuation calcifications in the caudate nuclei, putamina, globus pallidus, thalamus, and subcortical white matter. MRI denotes areas of calcification with hypointensity in gradient echo (GRE) or pulse sequences in susceptibility images (SWI).	[18]
CNS infections	Flaviviruses can affect the basal ganglia; however, it is generally much more asymmetrical.	[19]
CNS neoplasms	Primary CNS lymphoma primarily affects the thalamus and the basal ganglia but in heterogeneous and asymmetrical forms.	[20]

Treatment involves the administration of ethanol intravenously. This substance has a much higher affinity (up to 20 times greater) to alcohol dehydrogenase compared to methanol [2,4]. Thus, the attempt to treat this way is to reduce the production of format, which can also be potentiated by the administration of fomepizole or folinic acid [4].

## Conclusions

Metabolic acidosis and its causes (such as methanol intoxication) can lead to bilateral lesions in the basal ganglia and white matter in the brain. The clinical case reported in this article demonstrates neuroimaging lesions typical of metabolic acidosis, probably secondary to methanol intoxication. Promptly recognizing this clinical condition and its main differential diagnoses is crucial for the earlier establishment of the appropriate treatment for this group of patients in internal medicine services, emergency departments, and neurology services.
